# Considering family trees as a useful tool in family medicine: a systematic review

**DOI:** 10.1017/S1463423625000131

**Published:** 2025-02-27

**Authors:** Ksenija Tušek-Bunc, Alem Maksuti, Danica Rotar-Pavlič

**Affiliations:** 1 Department of Family Medicine, Faculty of Medicine, University of Maribor, Maribor, Slovenia; 2 Persuasion d.o.o., Ljubljana, Slovenia; 3 Department of Family Medicine, Faculty of Medicine, University of Ljubljana, Ljubljana, Slovenia

**Keywords:** family history, family medicine, family trees, systematic review

## Abstract

**Aim:**

The aim of this study was to perform a systematic literature review of the purpose, design, and use of family trees by family physicians (FPs).

**Background:**

Family trees offer a valuable contribution to understanding the relevance of the patient’s family history in comprehensive primary healthcare provision. There is little research on the role of family trees in the everyday practice of FPs. Studies often focus on specific diseases and their context: however, a comprehensive exploration of the usefulness of family trees is crucial for FPs.

**Methods:**

A systematic literature review was conducted through a keyword search in the PubMed and Cochrane databases. Based on the inclusion and exclusion criteria selected, 24 studies were identified and a qualitative analysis was performed.

**Findings:**

A total of 369 publications were identified across 32 fields. Twenty-four studies were included in the final analysis according to the QUOROM statement. The results underscore the role of family trees and highlight the value of this tool’s multidimensionality. The use of this tool directs FPs to consider a genetic cause and a possible referral to a geneticist. The value of a family tree lies in the personalized patient-oriented treatment in connection with hereditary risks for chronic diseases. For FPs, the greatest challenge in treating patients is determining their risk of developing a chronic disease or cancer. Using a family tree can improve the quality of their healthcare.

## Introduction

Family trees make a valuable contribution to understanding the relevance of the patient’s family history in comprehensive primary healthcare provision. Throughout history, family trees have offered an extraordinary source of information to improve the understanding and treatment of common chronic illnesses, including coronary heart disease, diabetes, various cancers, osteoporosis, and asthma (Keen *et al.*, 1999; Burke *et al.*, 2003; Harrison *et al.*, 2003; Kardia *et al.*, 2003; Ziogas and Anton-Culver, 2003; McGoldrick *et al.*, 2020). There is growing recognition that family trees also support tailored disease prevention, which may be more effective than existing approaches (Berg *et al.*, 2009). Family trees are often defined in medical literature as ‘family history’. The concept is grounded on the repeatedly verified hypothesis that similar diseases are based on common genetics. Taking a detailed family history represents the cornerstone of the genetic risk assessment, as it helps to ensure that important genetic information is not overlooked (Vance, 2020; Bylstra *et al.*, 2021). There is a wide variety of diseases that can be found in the medical literature. There are diseases that are inherited directly from parents and diseases that medical professionals believe are not transmitted from one generation to another. The medical literature discussing how similar diseases are based on common genetics argues that Alzheimer’s disease, Huntington’s disease, diabetes, cardiovascular disease, congenital hearing loss, iron overload, venous thromboembolism, chronic respiratory disease, schizophrenia, bipolar disease, antisocial personality disorder, depression, birth defects, and various malignancies have genetic components (Rich *et al.*, 2004; Dolan and Moore, 2007; Holt and Sly, 2009; De Hoog *et al.*, 2014; Dhiman *et al.*, 2014; Vaughn *et al.*, 2015; Al-Mamun *et al.*, 2016).

Family trees also have a social component. FPs have always asked patients about their family history to gain insight into their social and medical backgrounds. It helps them understand the context of the patient’s symptoms in terms of environmental and lifestyle causes of disease and the patient’s concerns about the nature of the illness (Emery and Rose, 1999; Dolan and Moore, 2007). Within the medical profession, especially in family medicine, there is a general consensus that medical care will be transformed profoundly as advances in understanding family trees (genetic, family, and social components) become incorporated into diagnosis, treatment, and prevention (Emery and Rose, 1999; Yoon *et al.*, 2002; Guttmacher *et al.*, 2004; Qureshi *et al.*, 2005; Emery *et al.*, 2007; Qureshi *et al.*, 2009; Yoon *et al.*, 2009; Flynn *et al.*, 2010; Mathers *et al.*, 2010; Williams *et al.*, 2011; Wilson *et al.*, 2012; Baer *et al.*, 2013; De Hoog *et al.*, 2014; Emery *et al.*, 2014; Ahmed *et al.*, 2016; Al-Mamun *et al.*, 2016; Nathan *et al.*, 2016).

Family trees are traditionally regarded as a routine part of obtaining the medical history, but it is not used in a systematic way in clinical practice (Langlands *et al.*, 2010; Emery *et al.*, 2010). This article reviews various approaches in the design and use of family trees in family medicine. It examines how this field of family medicine is regulated internationally and what Slovenia could learn from practices elsewhere. In addition, it examines problems with and/or barriers to the design and application of family trees, which have been pointed out by FPs. Therefore, it uses secondary analysis to obtain information from the systematic review performed (Gülpinar and Güçal Güçlü, 2013). The aim of this study is a systematic literature review of the purpose, design, and use of family trees in family medicine settings.

## Materials and methods

This study was conducted strictly in line with the PRISMA (Preferred Reporting Items for Systematic Reviews and Meta-Analysis) recommendations (Bolha *et al.*, 2021; Ho Man *et al.*, 2019).

### Literature search

Search strategies were developed and undertaken for the time period April 1999 to October 2019. Consistent with similar review studies (Bolha *et al.*, 2021; Ho Man *et al.*, 2019), a search engine was utilized to find scholarly literature related to family tree/history research. The Web of Science (WoS), an interdisciplinary electronic resource, was used as the relevant database. The English expressions *family*, *tree*, and *family history* were used as search terms. To find potential publications in Slovenian, the Cobiss system was used to search for results under Slovenian keywords.

Grading criteria were used to select the relevant articles. In the first step, the search results were reduced by filtering for articles posted in the last ten years and articles in English. The selected articles were then systematically reviewed. Original research that dealt with family trees was used as the inclusion criterion. Exclusion criteria included clinical case presentations, research protocols, columns, and opinions or comments. In the systematic review described, 24 articles were selected that met all the criteria (Table 1).

**Table 1.** Inclusion and exclusion criteria

Inclusion criteria	Exclusion criteria
Original empirical research studies Review research studies^*^ Comparative studies focused on tools, countries, or specific populations Evaluation studies and evaluations of tools	Non-research, expert studies Clinical case presentations Research protocols Columns Opinions

* Review studies are of utmost importance because family trees are analysed from the perspective of many research fields. Exporting them would risk overlooking some very important and high-quality studies.

### The QUOROM statement and qualitative analysis

After elimination by field of study, the selected publications were additionally systematically reviewed. The verified QUOROM statement for ‘improving the quality of reporting of meta-analysis’ was applied (Moher *et al.*, 1999) in the field selected. We used a verified checklist for standards for reporting meta-analysis. It is a holistic framework that includes an abstract, an introduction, methods, results, and a discussion/conclusion.

Because the field of research covers not only medical studies, only the original checklist (Moher *et al.* 1999) was slightly adapted. The checklist is organized into separate categories focused on the search, selection validity assessment, study characteristics, quantitative/qualitative data synthesis, and trial flow. It is presented in Table 2.

**Table 2.** Quality assessment checklist

Is the research question or study aim clearly stated?	Criteria: Yes No Not applicable (NA)
Is the study properly defined and conceptually clearly defined?	
Is the population in the study fully described? Is the sampling proper?	
Are the statistical methods described well?	
Are the results presented in a transparent and clear manner?	
Are the advantages and disadvantages of the research clear?	
Is the discussion rich in content and are the findings/conclusions clear?	
Overall quality rating	Criteria: good, fair, poor

Source: adapted from Moher *et al.* (1999, p. 1897).

The literature examining family trees and the significance of their use in family medicine was analysed in greater detail. In doing so, focus was placed on important segments (various categories) of family tree research that are primarily useful in family medicine. This yielded a comprehensive distribution of studies by group, according to the topic or the manner in which family trees are used in the studies.

## Results

### Basic information

The most general result obtained in the WoS database with the search string (((ALL = (family)) AND ALL = (history)) AND ALL = (tree))) was 4,558 publications. Because these were studies from 197 different fields, areas relevant to research on family trees/history in medicine were selected in the next step.

In the areas directly related to medicine, 369 publications were identified across 32 groups or fields. When selecting the fields, the criterion observed was that the field had to contain the word *medicine* and that it had to belong to a medical science.

**Figure 1.**
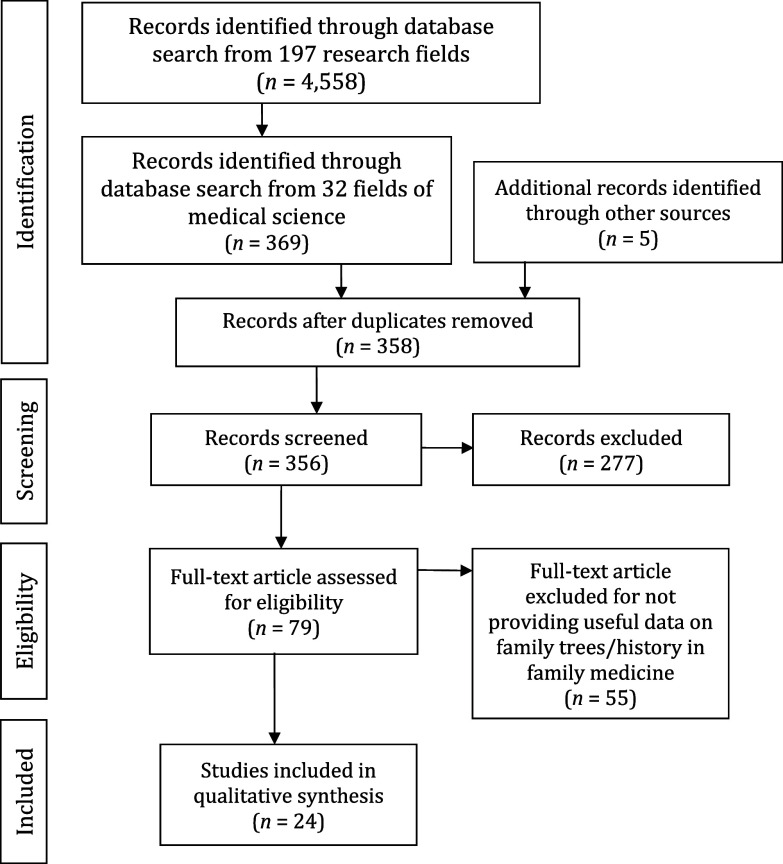
A PRISMA flow diagram of the literature review strategy applied.

The publication search and selection process is illustrated in Figure 1. After excluding publications from irrelevant fields, the authors focused on only 32 subfields of medicine. The screening process revealed 369 studies as relevant. After the review of titles and abstracts, 277 publications were excluded. In that way, 79 full studies were obtained, of which 55 were finally excluded due to unsatisfactory material and/or a lack of relevant data about the usefulness of family trees/history in family (primary care) medicine.

Ultimately, the systematic review identified 24 studies. These studies can be roughly divided into three groups. The first group deals with technological challenges in family tree design, the second group focuses on the possibility of setting up national databases, and the third group examines family physicians and their views on the feasibility of using family trees at the primary healthcare level.

### Main features of studies included in the systemic review

The main features of each registered study are reviewed in Table 3.

**Table 3.** Main features and quality assessment of studies included in the systematic review

Study	Country	Type/design	Subject	Method	Intended for	Main findings	Q_1_	Q_2_	Q_3_	Q_4_	Q_5_	Q_6_	Q_7_	OQ
Emery and Rose, 1999	UK	Review study	National family tree database	Secondary analysis	FPs	FH as a multidimensional tool makes it possible to examine patients’ concerns and explore the role of both nature and nurture in the aetiology and prevention of disease.	✓	✓	–	–	–	–	✓	F
Guttmacher *et al.*, 2004	USA	Review study	National family tree database	Secondary analysis	FPs, decision-makers	Use of modern tools – refining electronic means for gathering and analysing FH, further developing protocols that use EBM to craft individualized care guided by FH, and continuing to update the approach to FH.	✓	✓	–	–	–	–	–	F
Qureshi *et al.*, 2005	UK	Comparative study	Tool assessment	FHQ survey	FPs	FHQs identified most informants with genetic risks that are appropriately addressed: those with a FH of premature CHD, those warranting specialist referral, and those that might appropriately be offered carrier testing.	✓	✓	✓	✓	✓	✓	✓	G
Yoon *et al.*, 2002	USA	Review study	National family tree database	Secondary analysis	FPs	FH information in combination with other known risk factors could be used to provide more personalized information about the risk of common diseases, and it would lead to increased adoption of health-promoting behaviours.	✓	✓	✓	✓	✓	–	✓	G
Rich *et al.*, 2004	USA	Review study	New family tree tool	Secondary analysis	FPs	FH is the most important tool for diagnosis and risk assessment in medical genetics, and it promises to serve as a critical element in the use of predictive genetic testing.	✓	✓	✓	✓	✓	–	✓	F
Walter and Emery, 2005	UK	Qualitative study	Patient’s view of family tree	QCA	FPs, patients	Potential differences in the way patients and medical professionals assess and understand familial risk of cancer, heart disease, and diabetes. Eliciting the patient’s perspective when discussing the risk of chronic disease, particularly in the context of FH, could inform a more patient-centred approach to risk assessment and communication, and support patients in making informed decisions about the management of their disease risk.	✓	✓	✓	✓	✓	✓	✓	G
Walter and Emery, 2006	UK	Qualitative study	Patient’s view of family tree	QCA	FPs, patients	Factors influencing perceptions of FH may vary between individuals and between diseases. To use FH as a tool in preventive healthcare, the individual’s personal understanding of disease risk and ideas about the cause and controllability of a familial illness need to be considered. Perceived risk may then be used to motivate preventive health behaviours.	✓	✓	✓	✓	✓	✓	✓	G
Hall *et al.*, 2007	UK	Qualitative study	The importance of family trees	QCA	FPs, patients	Clinicians appeared to lack vocabulary to discuss FH in terms of capturing both genetic and environmental factors, and its relation to other risk factors. This created uncertainties for patients, and it carries potential clinical and social implications.	✓	✓	–	–	–	✓	–	P
Wood *et al.*, 2008	USA	Qualitative study	The importance of family trees	QCA	FPs, patients	Barriers to the effective application of cancer FH information included limitations of patients’ FH information, a lack of methods to systematically and efficiently focus on the most useful information, and a lack of accessible guidance for risk stratification and management.	✓	✓	–	–	–	✓	✓	F
Qureshi *et al.*, 2009	UK	Review study	Tool assessment	Secondary analysis	FPs	FH is a fundamental element of health information, and the ability to take an adequate and accurate cancer FH should be recognized as a core skill for all FPs, irrespective of the availability of tools.	✓	✓	✓	✓	✓	✓	✓	G
Yoon *et al.*, 2009	USA	Interdisciplinary study	Tool design	Mixed methods system	FPs	Developing Family Healthware, a new interactive, web-based tool that assesses familial risk for six chronic diseases and provides a ‘prevention plan’ with personalized recommendations for lifestyle changes and screening. Identifying effective FH tools and strategies that could be used to identify people at increased risk for chronic diseases and influence their health behaviours and use of preventive service.	✓	✓	✓	✓	✓	✓	✓	G
Flynn *et al.*, 2010	USA	Quantitative study	Tool assessment	Survey	FPs	Modifiable perception and resource factors independently associated with the quality of FH taking in a large and diverse sample of FPs. Improving FH quality to identify high-risk individuals will require multi-faceted interventions.	✓	✓	✓	✓	✓	✓	✓	G
Mathers *et al.*, 2010	UK	Qualitative study	The importance of family trees	QCA	FPs	The likely effectiveness of educational policy interventions aimed at FPs that focus solely on knowledge deficit models is questionable. There is a need to acknowledge how appropriate practice is constructed by FPs, within the context of accepted generalist roles and related identities.	✓	✓	✓	✓	✓	✓	✓	F
Williams *et al.*, 2011	USA	Qualitative study	FPs’ experiences with family trees	QCA	FPs	FPs’ experience with FH represents the synthesis of tensions between positive and negative experiences relating to process problems and the perception that FH must be collected.	✓	✓	✓	–	–	✓	–	P
Lim and Hewison, 2012	UK	Quantitative study	Medical staff experiences with family trees	Descriptive statistics	Medical staff	Misunderstanding and poor knowledge of cancer FH and the type of information required to create a FH even in a sample of people teaching and researching medicine and health issues. Public understanding of the value of family FH in cancer prevention and management is important if informed clinical decisions and appropriate health care are to be delivered.	✓	✓	✓	✓	✓	✓	✓	F
Wilson *et al.*, 2012	Canada	Interdisciplinary study	Tool assessment	Mixed methods system	FPs, medical staff	‘One size does not fit all’; there are several different uses to which FH information may be put, and FH tools have attributes that must vary according to these different purposes. A framework is proposed that points to the need for thoughtfulness about the components of tools beyond the FH information items and beyond a primary purpose of risk stratification. It is suggested that looking outside the field of genetics – for example, to cognitive psychology – may offer productive ways forward. Although one generic tool may not be capable of achieving all purposes, efficient platforms could be developed that facilitate multiple uses of FH information in primary care settings.	✓	✓	✓	✓	✓	–	✓	F
Baer *et al.*, 2013	USA	Quantitative study	Tool assessment	Mixed methods system	FPs	New documentation of a positive FH of cancer in coded EHR fields. Secondary outcomes included clinician reminders about screening and discussion of FH, lifestyle factors, and screening.	✓	✓	✓	✓	✓	✓	✓	F
Emery *et al.*, 2014	Australia	Quantitative study	Tool assessment	FHQ survey	FPs	FH screening questionnaire use in primary care as part of a systematic approach to tailored disease prevention.	✓	✓	✓	✓	✓	✓	✓	G
De Hoog *et al.*, 2014	Netherlands	Systematic review	Tool assessment: systematic review	Secondary analysis	FPs	The use of a FH tool improved the identification of patients at risk for disease.	✓	✓	✓	✓	✓	✓	✓	G
Dhiman *et al.*, 2014	UK	Cross-sectional study	Assessing availability and quality	Descriptive statistics	FPs	FH of CHD is documented in a small proportion of primary care records; where positive FH is documented, the details are insufficient to assess familial risk or populate CHD risk assessment tools. Data capture needs to be improved particularly for more disadvantaged patients, who may be most likely to benefit from CHD risk assessment.	✓	✓	✓	✓	✓	✓	✓	G
Ahmed *et al.*, 2016	UK	Qualitative study	Tool assessment	QCA	FPs	FPs’ concerns about the implementation of FH questionnaires as an intervention for identifying at-risk relatives.	✓	✓	✓	✓	✓	✓	✓	F
Chen *et al.*, 2016	USA	Qualitative study	National minority family tree database	QCA	FPs, Chinese minority	Limited usage of FH; cultural, distance, knowledge, and healthcare system–related barriers influenced FH use.	✓	✓	✓	✓	✓	✓	✓	F
Nathan *et al.*, 2016	UK	Review study	The importance of family trees	Secondary analysis	FPs, patients	FH use to develop personalized care plans and inform lifestyle choices to reduce FH-related risk.	✓	✓	–	–	–	–	–	P
Kim *et al.*, 2019	South Korea	Interdisciplinary study	National family tree database	Mixed methods system	FPs, decision-makers	Using the family tree database; prevalence rates for hypertension, diabetes, ischaemic heart disease, cerebrovascular disease, and cancer are higher for those with FH.	✓	✓	✓	✓	✓	✓	✓	G

*Note*: The studies are arranged in chronological order; FH = family history; EBM = evidence-based medicine; FHQ = family history questionnaire; CHD = coronary heart disease; QCA = qualitative content analysis; Q_1_ = Is the research question or study aim clearly stated?; Q_2_ = Is the study properly defined and conceptually clearly defined?; Q_3_ = Is the population in the study fully described? Is the sampling proper?; Q_4_ = Are the statistical methods described well?; Q_5_ = Are the results presented in a transparent and clear manner?; Q_6_ = Are the advantages and disadvantages of the research clear?; Q_7_ = Is the discussion rich in content and are the findings/conclusions clear?; OQ = Overall quality rating (P = Poor, F = Fair, G = Good).

The trend is encouraging and in line with the assumptions or the goal of this research. The studies included in the review (Table 3) were published between 1999 and 2019. Altogether, 24 studies have reported on attempts to establish national family tree databases, and they drew attention to the importance of family trees at the primary healthcare level. The studies also emphasized the importance of evaluating tools for making family trees by FPs, and they revealed the FPs’ views on the importance of using family trees at primary care clinics.

The studies analysed have different methodological approaches. In addition to traditional positivist and comparative studies, there are also qualitative studies. In the latter, researchers deal with issues related to family trees inductively, from the bottom up, and in this way try to create paradigmatic models to justify the use of family trees in medicine and other fields. Such an approach is probably also the greatest added value in research on an under-researched area.

In the second part, a composite coder assessed the quality of the studies analysed. Although each study is an important part of the research puzzle, the quality of the research varies. The results of the analysis based on the previously designed checklist are presented in Table 3.

The results of the qualitative assessment of the studies included in the analysis showed that the majority of the studies were conducted correctly. Moreover, most of the studies could be rated as good because they meet all the research criteria, which is also confirmed by the journals the studies are published in. In addition to being set up correctly, these studies are also methodologically grounded, the populations are clearly defined, the sampling is correct, and the results are transparent.

Certain studies (Hall *et al.*, 2007; Williams *et al.*, 2011; Nathan *et al.*, 2016) have clearly defined objectives but lack a well-defined methodological approach. Moreover, the same studies are flawed by conclusions that are not described as clearly as possible. Some studies, although we rated them as good, do not have the advantages and disadvantages (limitations) listed. However, we considered this to be a less important feature.

The assessment of the quality of the selected studies follows the methodology set out in the introduction on a systematic review of the literature. On the other hand, a systematic review of the literature should offer a certain typology that would classify the available studies according to their content and object of research. Only in this way will the potential and quality of the studies analysed, which deal with family trees from different perspectives, become apparent.

With this aim, in the discussion (below), in interaction with the ideas of the best-quality studies included in our analysis, we highlight a typology that includes four categories. The first group of studies focuses on tools and technologies for collecting and managing family trees in medicine; the second focuses on the need to establish national databases; the third focuses on FPs’ views and understanding the potential of family trees; and the fourth raises ethical, legal, and social questions related to the creation of family trees and their use in medical practice.

## Discussion

### Tools and technology

The creation of family trees involves various tools, such as face-to-face interviews and medical history questionnaires, and obtaining or confirming genealogical information, sometimes with the involvement of genetics experts. FPs are primarily in charge of designing family trees. This process is often time consuming because it requires commitment, meticulousness, and entering data into a database (Emery and Rose, 1999; Rich *et al.*, 2004; De Hoog *et al.*, 2014).

Over the past decade, increasingly more studies have emerged in which researchers investigate how to use technology to make it easier for FPs to prepare family trees (Qureshi *et al.*, 2005; Qureshi *et al.*, 2009; Scheuner *et al.*, 2009; Wilson *et al.*, 2012; Baer *et al.*, 2013; De Hoog *et al.*, 2014). De Hoog *et al.* (2014) identified 18 different family history tools and offered a holistic taxonomy that refers to the types of diseases for which family trees are used, the healthcare levels in which they are used (primary, secondary, etc.), and the degree of computerization/digitization of a particular tool.

Qureshi *et al.* (2005) developed a similar taxonomy in their study. They also identified 18 family history tools, with 11 developed for use in primary care. In addition to the fact that some of the tools are based on a CSAQ (computerized self-administered questionnaire) survey approach, virtually no family history tools provide an electronic database that would be compatible with other medical databases. In an era of widespread digitization, this weakness should be eliminated, which has been pointed out by other healthcare researchers (e.g., Menvielle *et al.*, 2017).

Baer *et al.* (2013) offered a web application in which patients enter data themselves. The Your Health Snapshot (YHS) tool is a patient-administered, web-based risk appraisal tool that was constructed using a risk assessment framework. More than a family tree, YHS is a tool that ‘assigns relative risk estimates to environmental, dietary, and lifestyle factors based on relevant epidemiologic studies’. A similar web-based tool was also developed in 2004 by the US Centers for Disease Control and Prevention (US CDC; Yoon *et al.*, 2009).

Family tree tools are frequently used to collect information that is not directly related to the family tree. This was pointed out by Wilson *et al.* (2012), who found that, although one generic tool is not capable of achieving all purposes (especially not at all levels of healthcare), there is a need for efficient platforms that facilitate multiple uses of family history information in primary care medicine. This is widely discussed among researchers dealing with national family tree databases, which is addressed in the next section.

### National family tree databases

National family tree databases should become part of routine practice in family medicine. The idea of a national database system of family trees is not new in primary healthcare medicine. Studies in which researchers have pointed to the importance of national databases date back to the 1990s (Kinmonth *et al.*, 1998; Emery and Rose, 1999). These studies describe several attempts to create a national database of family trees at the primary healthcare level. Similar contributions are also found today (Emery *et al.*, 2014; Kim *et al.*, 2019).

South Korea has a national social health insurance system, which reached universal coverage of all Korean residents in 1989 (Kim *et al.*, 2019). In addition to the demographic, socioeconomic, and disability registration information, this system includes a wide range of health risk factors, it facilitates national health screening programmes (general checkups, cancer screening, etc.), and it provides detailed information on the healthcare utilization of all insured residents. The key result of this system is the National Health Information Database (NHID), which was created to meet various demands for data. Because the NHID has some limitations, Kim *et al.* (2019) developed a special code system to logically convey interpersonal relationships within families and establish a database of interpersonal family relationships of the entire population.

The South Korean attempt and similar ones, of course, were conditional on the development of technology that determines the development of various subfields of family medicine (Jenkins and Oyama, 2020). A common problem that virtually all researchers point out is the incoherence and incompatibility of family trees with other medical databases in the country. De Hoog *et al.* (2014) found that no family tree/history tool (out of 18 examined) allows for the electronic transfer of family tree information to electronic medical record systems. Researchers have reported similar findings in the case of Australia (Emery *et al.*, 2014).

The existence of a national database depends on the institutional structure of the health and social security system in the country. In countries with underdeveloped health infrastructure (e.g., public institutions, insurance companies, and support infrastructure), it is unrealistic to expect there to be a national family tree database at the primary healthcare level (De Hoog *et al.*, 2014). On the other hand, the creation of a national system requires the goodwill of FPs, who are the primary sources of information about patients and their families. Making family trees requires resources and time that FPs do not usually have (Williams *et al.*, 2011). Sometimes there are also other barriers, which are discussed below.

Slovenia has no national family tree database at the primary healthcare level. Moreover, the present study is the first to highlight the untapped potential of family trees for the development of family medicine in Slovenia. In the past, only some bachelor’s and master’s theses have been written, in which the importance of family trees in the FPs’ treatment of chronic diseases was highlighted (Praprotnik, 2014).

### Family physicians’ perspective

Most studies examining FPs’ views on the creation and use of family trees are based on a qualitative approach. Researchers conduct individual and group interviews (focus groups), in which FPs are asked a wide variety of questions about family trees and their use in primary medicine (Walter and Emery, 2006; Wood *et al.*, 2008; Mathers *et al.*, 2010; Williams *et al.*, 2011). These studies can be roughly divided into two groups: 1) studies discussing the increasing usability of family trees in primary medicine, and 2) studies in which FPs point out the difficulties in and barriers to creating a (national) family tree database.

Mathers *et al.* (2010) found that family trees (and genetic concepts more generally) are clearly part of current family practice in the UK. It was once the case that FPs solely looked ‘for social and psychological influence via the family history’. This has changed; today’s practice is different. According to Mathers *et al.* (2010), for British FPs a family tree is more than a diagnostic/risk assessment tool. It exists within the wider concept of the family doctor and offers insight into other facets of patients and families. Researchers from other countries and/or specific ethnic groups have also come to the same conclusion (Hall *et al.*, 2007; Wood *et al.*, 2008; Williams *et al.*, 2011; Chen *et al.*, 2016).

In general, the positive experiences of FPs are associated with the versatile usefulness of family trees. On the other hand, regarding difficulties in creating (and using) a family tree database, FPs have highlighted the following issues: the fact that family tree data needs to be collected, which is time consuming; confusion about the use of family trees; perceived inaccuracies and incompleteness of the information provided; and the personal liability of FPs, which contributes to a negative experience (Williams *et al.*, 2011).

However, some systemic features should be highlighted as central barriers to creating a national family tree database. It is often overlooked that FPs do not decide to design family trees. In Slovenia, for example, doctors are part of the public health system, which is mostly financed through the national health insurance system, which does not provide adequate motivation for innovation. This means that FPs are paid to treat patients and not to perform activities that require additional time and work (e.g., creating family trees). The FPs interviewed in other countries expressed the same concerns (Hall *et al.*, 2007; Mathers *et al.*, 2010; Williams *et al.*, 2011).

Family history has garnered increased attention as a means to help clarify a person’s risk of developing common chronic conditions, such as diabetes mellitus, coronary heart disease, asthma, and certain types of cancer. That is why interest in researching and developing family trees is growing. The medical profession agrees on the importance of family trees as a valuable source of data for understanding and treating patients. Virtually all the authors cited suggest that family trees are a tool that all countries should have in the digital age. The literature also acknowledges the importance of national family tree databases that are compatible with other digital medical registries (De Hoog *et al.*, 2014). However, there are some obstacles and barriers in the field. Data are collected across many models and with many tools, which means databases are often incompatible with one another (Qureshi *et al.*, 2005). The burden of creating family tree databases in most countries is tied to the primary healthcare level. FPs collect and communicate information they receive from patients through interviews or pre-designed questionnaires. Because this work is indeed time consuming and, in most cases, unpaid, the databases are often deficient, or they do not exist at all.

Despite recent insights into the importance of family trees and family tree databases in primary care, there appear to be substantial barriers to developing family trees and creating databases, including overburdened doctors, inadequate payment systems and reimbursement policies for FPs, current modes of organizing family medicine practices, varying patient expectations, inaccurate information reported by patients, a lack of proper training to collect and interpret family trees, and a lack of FPs’ own knowledge and skills. FPs may neglect family history because of the amount of time required to collect the information. They were likely to report ‘lack of time during the visit’ as a barrier. Certainly, FPs typically spend far less time obtaining family tree information than suggested by family medicine experts. Overcoming the problem of the time and effort required for creating and analysing family trees remains a daunting challenge (Rich *et al.*, 2004).

## Ethical, legal, and social issues

Furthermore, family trees raise issues of ethical and legal implications, including privacy, confidentiality, data ownership, and informed consent. FPs should be aware of the ethical, legal, and social implications of collecting family tree information, particularly in the current climate of uncertainty about the privacy of medical information. Various legal issues can affect the collection of family history information under some circumstances, including informed consent, data ownership and protection, obligation to disclose data, and reporting requirements. In addition, the potential negative outcomes of assessing family history must be considered carefully. For example, limited information has been obtained about stigmatization, discrimination, privacy/confidentiality, and personal, family, and social issues associated with family history assessment and risk labelling (Lucassen *et al.*, 2006). Labelling a person as high or moderate risk for disease may have important psychological, social, and economic costs. The use of a family history tool for public health purposes could only be successful if people perceived greater benefit than risk associated with revealing family medical information, if there was no stigma associated with being at above-average risk, and if there were interventions and options for behaviour change that could make a difference in reducing morbidity and mortality (Yoon *et al.*, 2002).Although most FPs are aware of the potential for fatalism, anxiety, impairment of self-image, depression, or blame associated with collecting family history information, no data are available to suggest that these unintended behaviours or feelings do in fact occur or, if they do, how common they are. This is another aspect of obtaining family trees that requires further research (Yoon *et al.*, 2009).

Both researchers and FPs agree that the issue of family tree databases needs to be regulated at the systemic level. Certain countries can provide best practice examples (Kim *et al.*, 2019). In fact, the creation of national family tree databases requires a holistic and sustainable approach (Nardi *et al.*, 2013) that will justify the initial costs of creation and clearly highlight the benefits that doctors, patients, and their relatives can derive from this. Only then can the second step take place, the selection of a model that may be eclectic but validated at the national level. Therefore, joint action by decision-makers, the healthcare system, responsible institutions, doctors, and patients (a group not addressed in this study; Hall *et al.*, 2007) is required.

## Conclusion

Family trees should make their way into family practice as a routine procedure with their own qualities. Family trees are a tool that can significantly contribute to better patient care. A review of experiences from other countries has revealed some challenges. Designing family trees is a demanding task that should not be delegated only to FPs. Therefore, there is a need to create a national family tree database through the collaboration of FPs and relevant health institutions. The creation of a national database would require the involvement of all stakeholders, including FPs, politicians, and technical staff to set up the system operationally. The versatility of using a national family tree database is confirmed by virtually all studies that were analysed in detail. A national database would be an extremely useful tool for the development of family medicine.
